# Military Surgery

**Published:** 1917-07

**Authors:** 


**Military Surgery.** By DUNLOP PEARLY PENHALLOW, S.B., M.D. (Hart.). Pp. xiv. 432. Oxford Medical Publications. 1916. Price 15s. net.

This is an interesting book on war surgery, but it is by no means an exhaustive treatise on the subject. If we turn to any particular subject, we are apt to feel disappointment at the extent of the information given about it. There seems so much more that might have been written with advantage, and here and there we feel strongly disinclined to agree with what the author does state. Take as an example of incomplete information the subject of injuries of blood vessels and aneurysm. There is no reference in the list of recent literature consulted to either Sir Wm. Makin's very valuable contribution on "The vascular lesions produced by gun-shot injuries, and their results" (*British Journal of Surgery*, January, 1916), or to the very valuable collection of records of traumatic aneurysms published in earlier numbers of this journal. However, many quite interesting and instructive cases are related; and the illustrations of cases, and the use of apparatus, and the reproduction of skiagrams are very good and helpful.

The chief interest of this book seems to us to be the experience of the author in plating gun-shot fractures. This has so often proved disastrous, that it has been very strongly condemned. It has been the experience of surgeons who have used this method in gun-shot fractures that very extensive necrosis was caused by it. Perhaps this has been due to the enormous plates and long screws we have seen provided as a part of army surgical equipment. As the author points out, a certain amount of necrosis is very apt to follow suppurating gun-shot fractures which are not plated, and he does not think plating materially increases the likelihood of this. His results seem certainly to justify his practice, and appear to us sufficient reason for a wider adoption of it. He has plated fifteen cases "with no failures and with excellent functional results." He lays great stress on the need for providing drainage of the medullary cavity of the plated bone, and he removes the plate as soon as union takes place. Several instructive cases are given illustrating the treatment, and some important matters, in the technique, besides drainage of the medulla, are referred to. The advantage of fixing fragments, which are difficult to bring in position, in this way is sufficiently obvious to make the method very acceptable to the military surgeon, if it is not followed by the disastrous consequences of which surgeons have been afraid We note that the author recommends constant irrigation with Eusol or saline after the operation, until the infection clears up. But the recent results of Carrel and other surgeons abroad, who have adopted his method of sterilising wounds, have been so excellent, that these surgeons have been able to plate gun-shot fractures thus sterilised with the best results.

We are disappointed with the author's description of the treatment of gun-shot fractures of the knee-joint. He is helpful in his account of the best method of dealing with the recent wounds, as based on the experience of Col. Gray and Capt. Lockwood, but we get almost no information as to how to deal with serious infection of the joint, which is one of the most disastrous conditions met with in military surgery, and demands prompt and energetic treatment, nor is the question of primary amputation considered.

His book is quite worth reading, but it can hardly be used as a work of reference to be consulted for help in dealing with difficult cases of military surgery.

